# 548 Outcomes of Minimally Invasive Excision, Epidermal Autografting, and Skin Substitute in Adult Mid-deep Dermal Burns

**DOI:** 10.1093/jbcr/iraf019.177

**Published:** 2025-04-01

**Authors:** Kaitlyn Malek, Tony Zhao, Richard Lou, Anjay Khandelwal

**Affiliations:** Northeast Ohio Medical University; Northeast Ohio Medical University; Akron Children’s Hospital; Adult and Pediatric Burn Institute at Akron Children’s

## Abstract

**Introduction:**

The standard of care (SOC) for adult mid-deep dermal burns typically involves conservative management with delayed surgery if indicated. This study evaluates the outcomes of Minimally invasive excision with Epidermal autografting and Poly-lactic acid skin substitute (MEP) compared to SOC.

**Methods:**

A retrospective review of adult burn patients from 2021 to 2024 undergoing MEP for mid-deep dermal burns compared to a propensity-matched SOC group (matched by age, TBSA, and injury mechanism) from 2017-2020. Outcomes included length of stay (LOS), 90%+ healed burns by post-operative or post-burn day ten, number of sedations required and infections. Financial outcomes utilized actual patient charges, cost:charge ratio and payments received. Descriptive statistics were used for demographics and univariate regression analyses were used to assess MEP’s impact on LOS, sedations, infections and time to healing.

**Results:**

There were 44 patients that met inclusion criteria, of which 39 were propensity matched to a SOC group. No differences in age, sex, %TBSA and etiology. MEP patients had a longer median length of stay (4.0 vs. 2.0 days, p=0.008) and required more sedations (median of 1vs 0, p = < 0.001). There was a significant difference in the number of infections that occurred as no infections occurred in the MEP group compared to 23.1% in SOC (p=0.002). By day 10, 82.1% of MEP patients achieved ≥90% healing compared to 38.5% of SOC (p=0.001). Lastly, the total charges, as anticipated, were higher in the MEP group (median $84,982.51 vs. $15,524.34, p< 0.001). In the SOC group, 42% of charges were paid and 46% in the MEP group.

Univariate regression analysis was then utilized to better assess the relationship between MEP as a single predictor variable and each outcome measure, which demonstrated that MEP was not significantly associated with LOS (p = 0.26) or number of sedations required (p=0.37). However, the analysis demonstrated MEP patients were five times more likely to achieve ≥90% healing (p=0.002) by post-operative/burn day ten.

**Conclusions:**

MEP patients were five times more likely to achieve ≥90% healing by day ten and had no infections while not significantly affecting LOS or sedations required. As expected, charges were higher in the MEP group, however, with a higher percentage of charges paid, MEP is financially sustainable. MEP represents a viable alternative to traditional, conservative methods of burn care for mid-deep dermal burns in adult patients.

**Applicability of Research to Practice:**

Integration of MEP into clinical practice could lead to a shift in burn management that prioritizes quality and service, while being financially sound.

**Funding for the Study:**

N/A

